# Distorted Low-Level Visual Features Affect Saliency-Based Visual Attention

**DOI:** 10.3389/fncom.2016.00124

**Published:** 2016-11-29

**Authors:** Hamed Bahmani, Siegfried Wahl

**Affiliations:** ^1^ZEISS Vision Science Lab, Institute for Ophthalmic Research, Eberhard Karls Universität TübingenTübingen, Germany; ^2^Physiology of Cognitive Processes, Max Planck Institute for Biological CyberneticsTübingen, Germany; ^3^Bernstein Center for Computational NeuroscienceTübingen, Germany

**Keywords:** visual attention, saliency, distortion, low-level features, computational modeling

## Introduction

Image distortions can attract attention away from the natural scene saliency (Redi et al., 2011). Performance of viewers in visual search tasks and their fixation patterns are also affected by different types and amounts of distortions (Vu et al., 2008). In this paper, we have discussed the opinion that distortions could largely affect the performance of predictive models of visual attention, and simulated the effects of distorted low-level visual features on the saliency-based bottom-up visual attention. Saliency is a fast and pre-attentive mechanism for orienting visual attention to intrinsically important objects which pop-out more easily in a cluttered scene. Distortion of the low-level features that contribute to saliency may impair the readiness of the visual system in detection of salient objects, which may have major implications for critical situations like driving or locomotion. These distortions in natural life can be introduced by eye diseases such as cataract, or spectacles which may alter color perception (de Fez et al., 2002) or cause undesired optical effects like blurring, non-uniform magnification, and image displacement (Barbero and Portilla, 2016). The extent to which each of these distorted saliency features may affect the attentional performance is addressed in this paper by employing a biologically-inspired predictive model of visual attention. We briefly summarize the current standing of computational work on visual attention models in the following section and suggest a simple and influential model of saliency to examine the above hypothesis. Furthermore, we demonstrate in an example the hindered performance of the predictive saliency model on distorted images.

## Models of visual attention

Despite the widely shared belief in general public that we see everything around us, only a small fraction of the information registered by the visual system reaches levels of processing that mediate perception and directly influence behavior (Itti and Koch, 2000). Selective attention is the key to this process which turns looking into seeing (Carrasco, 2011). But how does the visual system select and enhance the representation of one particular feature or spatial location over less relevant features and locations? Much evidence has accumulated in favor of a two-component framework for the control of where in a visual scene attention is deployed: a bottom-up, fast, and image-based mechanism that biases the observer toward selecting stimuli based on their saliency, and a second slower, top-down mechanism, which uses task-dependent cues to direct the spotlight of attention under voluntary control. Koch and Ullman (1985) introduced the idea of a saliency map to accomplish pre-attentive selection. A saliency map is an explicit two-dimensional map that encodes the saliency of visual objects in the environment purely based on the low-level visual attributes of the object (Itti et al., 1998). Competition among neurons in this map gives rise to a single winning location that corresponds to the most salient object, which constitutes the next target. If this location is subsequently inhibited, the system automatically shifts to the next most salient location. This internal dynamic models the saccadic eye movements in visual search. This purely computational hypothesis received experimental support from many electrophysiological studies including single-cell recordings from lateral intraparietal neurons of macaque monkeys which responded to visual stimuli only when those stimuli were made salient (Gottlieb et al., 1998).

Today, more than fifty quantitative models for saliency and fixation prediction are available which have been accumulated over the past 20 years; some of them tried to also incorporate top-down attention (Borji and Itti, 2013; Borji et al., 2013) or context-aware saliency detection (Goferman et al., 2012). However, not all of them are biologically plausible (Zhang and Sclaroff, 2013) or explain low-level visual features (Kümmerer et al., 2014); and the metrics used to compare the performance of these models are often different and inconsistent with each other (Kümmerer et al., 2015; Gide et al., 2016). For the purpose of this paper, we chose the original model of saliency-based visual attention for rapid scene analysis by Itti et al. (1998) for its utmost biological plausibility and simple dichotomy between low-level visual features which allows us to look at different distortion effects on each feature map independently, as well as the final saliency map. In the next section, we explain how this model can be used to simulate the effect of distortions on visual attention and visual search.

## Attention and image distortions

Visual attention models have been used in many computer vision applications (Pal, 2016), including image and video compression and retrieval (Ouerhani et al., 2001; Li et al., 2011), multimedia technologies (Le Callet and Niebur, 2013), and 2D (Akamine and Farias, 2014) and 3D (Jiang et al., 2014) image quality assessment. Moreover, researchers have conducted eye-tracking experiments to judge image quality instead of using a computational model for visual attention, thus making the results independent of the reliability of an attention model (Vu et al., 2008; Liu and Heynderickx, 2011; Redi et al., 2011). A very recent paper (Gide et al., 2016, available online) has evaluated several state-of-the-art visual attention models over multiple databases consisting of distorted images with various types and levels of distortions. However, types of distortions that have been addressed in this paper (e.g., blur, noise and compression) are different from the low-level features taken into account by our selected model (intensity, color, and orientation); and are rather important from image processing points of view such as image acquisition, compression and transmission. In this opinion paper, we suggest a biologically plausible model for a similar purpose, which is applicable to more clinical and behavioral studies like simulating eye diseases or effects of eyewear. It can be also used to study the effect of distortions on visual attention in natural circumstances like driving and sports.

**Figure 1:**
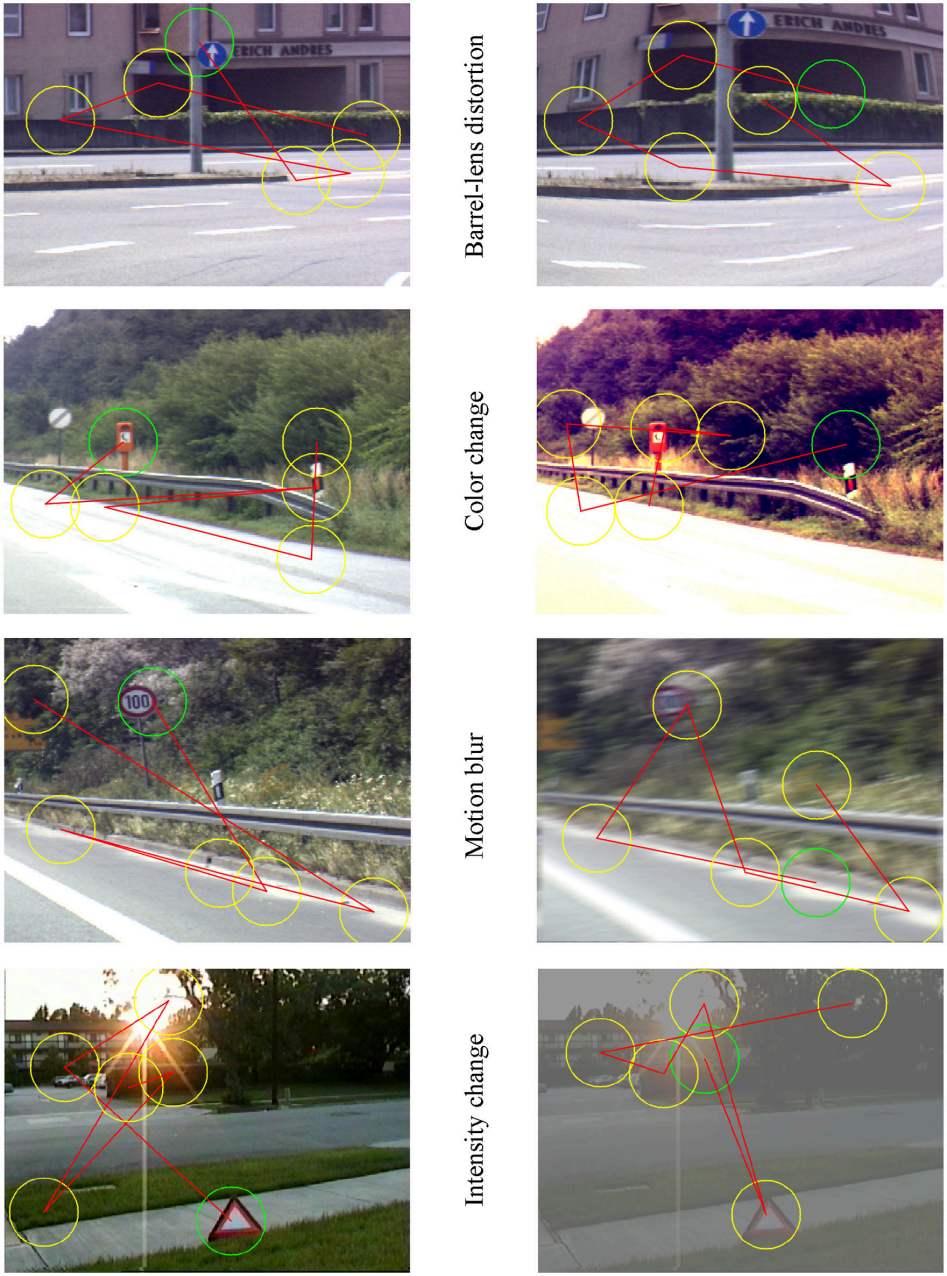
**Natural images consisting of one or more traffic signs in a cluttered scene and their simulated distorted versions are fed to a bottom-up visual attention model predicting the performance of human vision as the number of simulated saccades before detecting the target**. Green circle in all panels indicates the “first hit” predicted as the most salient location or object by the model, while yellow circles, and the path among them show next *salient* spots on the image and the scan path respectively (*simulated saccades*). From the top to the bottom, right panels are the barrel-lens aberration distortion, altered color, motion blurred, and altered intensity/contrast version of the corresponding left panel.

As a demonstration of the proposed opinion, we simulated an attention-demanding detection task while driving in a purely computational environment. We tested the model of bottom-up visual attention for the effects of distortions on a traffic sign search task, using manipulated static images containing different traffic signs in cluttered scenes. If a stimulus like traffic sign is sufficiently salient, it will pop out of the visual scene. The speed of this saliency-based form of attention is on the order of 25–50 ms per item (Itti and Koch, 2001a) which is sufficiently fast for tasks like driving. We used a simple performance measure similar to Bahmani et al. (2008) to quantify the goodness of the model in detecting targets on distorted images. We compared the performance of the model on natural and distorted images in Figure 1 by illustrating the number of false hits before detecting the target. We modified four original images (left panels) containing one or more traffic signs in a natural cluttered scene from a traffic sign image database (Itti and Koch, 2001b; available on Itti's Lab homepage: ilab.usc.edu) to obtain simulated barrel-lens-aberration distortion, manipulated color contrast, motion-blurred distortion, and distorted contrast/intensity versions (right panels). We then fed these images to the Saliency model (Walther and Koch, 2006; available online at: www.saliencytoolbox.net) and compared the performance of the visual search on original and distorted images. The model does not need a training phase or parameter setting and generates the simulated saccadic path of human observer instantly. As already noticeable in Figure 1, the main target (inside the green circle on the left panels) has been either not detected, or detected late after a few saccades on distorted images (see scan paths on the right panels). In all examples the visual search for the target starts with the green circle which indicates the first hit, and continues up to six points on the image creating a scan path which simulates saccades.

## Conclusion

The ability to direct the gaze as fast as possible toward salient objects in cluttered scenes has evolutionary significance because it allows the animal to detect quickly prey and predators in the visual world. In modern human era, we are not concerned much about predators, but our daily life and social interactions rely heavily on a rapid and reliable visual processing. Impairment of a fast pre-attentive mechanism for visual search has major implications in situations like driving, where rapid reaction to visual stimuli is important. In this paper, we proposed the use of a predictive model to evaluate the effects of distorted early visual features on saliency. Furthermore, we provided examples of distorted natural images with altered colors, orientation and contrast components and tested the performance of a simulated visual search task driven by bottom-up visual attention mechanism. Our results confirm a substantial effect of distorted early visual features on detectability of salient objects in natural cluttered scenes. These effects should be taken into account in situations which high levels of visual attention is required. Moreover, we suggest further experiments to compare the performance of computational models of visual attention with human observers to confirm the results of this simulation, or discover differences in robustness of biological and computational visual systems to distortions.

## Author contributions

HB and SW conceived the opinion and wrote the paper.

## Funding

The work was supported by the Eberhard-Karls-University Tuebingen as part of the German Excellence Initiative from the Federal Ministry of Education and Research (BMBF).

### Conflict of interest statement

The authors declare that the research was conducted in the absence of any commercial or financial relationships that could be construed as a potential conflict of interest. The reviewer MZ and handling Editor declared their shared affiliation, and the handling Editor states that the process nevertheless met the standards of a fair and objective review.

## References

[B1] Akamine W. Y. L. Farias M. C. Q. (2014). Incorporating visual attention models into video quality metrics, in 90160O (San Francisco).

[B2] Bahmani H. Nasrabadi A. M. Gholpayeghani M. R. H. (2008). Nonlinear data fusion in saliency-based visual attention, in 2008 4th International IEEE Conference Intelligent Systems, IS 2008 (Varna), 327–330.

[B3] Barbero S. Portilla J. (2016). The relationship between dioptric power and magnification in progressive addition lenses. Ophthalmic Physiol. Opt. 36, 421–427. 10.1111/opo.12301 27146008

[B4] Borji A. Itti L. (2013). State-of-the-art in visual attention modeling. IEEE Trans. Pattern Anal. Mach. Intell. 35, 185–207. 10.1109/TPAMI.2012.89 22487985

[B5] Borji A. Sihite D. N. Itti L. (2013). Quantitative analysis of human-model agreement in visual saliency modeling: a comparative study. IEEE Trans. Image Process. 22, 55–69. 10.1109/TIP.2012.2210727 22868572

[B6] Carrasco M. (2011). Visual attention: the past 25 years. Vision Res. 51, 1484–1525. 10.1016/j.visres.2011.04.012 21549742 PMC3390154

[B7] de Fez M. D. Luque M. J. Viqueira V. (2002). Enhancement of contrast sensitivity and losses of chromatic discrimination with tinted lenses. Optom. Vis. Sci. 79, 590–597. 10.1097/00006324-200209000-00010 12322929

[B8] Gide M. S. Dodge S. F. Karam L. J. (2016). The Effect of Distortions on the Prediction of Visual Attention. Vol. 53 Available online at: http://arxiv.org/abs/1604.03882

[B9] Goferman S. Zelnik-Manor L. Tal A. (2012). Context-aware saliency detection. IEEE Trans. Pattern Anal. Mach. Intell. 34, 1915–1926. 10.1109/TPAMI.2011.272 22201056

[B10] Gottlieb J. P. Kusunoki M. Goldberg M. E. (1998). The representation of visual salience in monkey parietal cortex. Nature 391, 481–484. 10.1038/35135 9461214

[B11] Itti L. Koch C. (2000). A saliency-based search mechanism for overt and covert shifts of visual attention. Vision Res. 40, 1489–1506. 10.1016/S0042-6989(99)00163-7 10788654

[B12] Itti L. Koch C. (2001a). Computational modelling of visual attention. Nat. Rev. Neurosci. 2, 194–203. 10.1038/35058500 11256080

[B13] Itti L. Koch C. (2001b). Feature combination strategies for saliency-based visual attention systems. J. Electron. Imaging 10, 161–169. 10.1117/1.1333677

[B14] Itti L. Koch C. Niebur E. (1998). A model of saliency based visual attention for rapid scene analysis. IEEE Trans. Pattern Anal. Mach. Intell. 20, 1254–1259. 10.1016/S1053-5357(00)00088-3

[B15] Jiang Q. Duan F. Shao F. (2014). 3D visual attention for stereoscopic image quality assessment. J. Softw. 9, 1841–1847. 10.4304/jsw.9.7.1841-1847

[B16] Koch C. Ullman S. (1985). Shifts in selective visual attention: towards the underlying neural circuitry. Hum. Neurobiol. 4, 219–227. 10.1016/j.imavis.2008.02.004 3836989

[B17] Kümmerer M. Theis L. Bethge M. (2014). Deep gaze I-boosting saliency prediction with feature maps trained on ImageNet, in arXiv1411.1045 [cs, q-bio, stat]. Available online at: http://arxiv.org/abs/1411.1045\nfiles/1004/arXiv-Kummerer_et_al-2014-Deep_Gaze_I-Boosting_Saliency_Prediction_with_Feature_Maps_Trained_on_ImageNet.pdf

[B18] Kümmerer M. Wallis T. S. A. Bethge M. (2015). Information-theoretic model comparison unifies saliency metrics. Proc. Natl. Acad. Sci. U.S.A. 112, 16054–16059. 10.1073/pnas.1510393112 26655340 PMC4702965

[B19] Le Callet P. Niebur E. (2013). Visual attention and applications in multimedia technologies. Proc. IEEE Inst. Electr. Electron Eng. 101, 2058–2067. 10.1109/JPROC.2013.2265801 24489403 PMC3902206

[B20] Li Z. Qin S. Itti L. (2011). Visual attention guided bit allocation in video compression. Image Vis. Comput. 29, 1–14. 10.1016/j.imavis.2010.07.001

[B21] Liu H. Heynderickx I. (2011). Visual attention in objective image quality assessment: based on eye-tracking data. IEEE Trans. Circuits Syst. Video Technol. 21, 971–982. 10.1109/TCSVT.2011.2133770

[B22] Ouerhani N. Bracamonte J. Hugli H. Ansorge M. Pellandini F. (2001). Adaptive color image compression based on visual attention, in Proceedings of the 11th International Conference on Image Analysis and Processing (Palermo), 416–421.

[B23] Pal R. (ed.). (2016). Innovative Research in Attention Modeling and Computer Vision Applications. Hershey, PA: IGI Publishing 10.4018/978-1-4666-8723-3

[B24] Redi J. Liu H. Zunino R. Heynderickx I. (2011). Interactions of visual attention and quality perception. Int. Soc. Opt. Photonics. 7865, 78650S–78650S-11. 10.1117/12.876712

[B25] Vu C. T. Larson E. C. Chandler D. M. (2008). Visual fixation patterns when judging image quality: effects of distortion type, amount, and subject experience, in 2008 IEEE Southwest Symposium on Image Analysis and Interpretation (Santa Fe, NM: IEEE), 73–76. 10.1109/SSIAI.2008.4512288

[B26] Walther D. Koch C. (2006). Modeling attention to salient proto-objects. Neural Netw. 19, 1395–1407. 10.1016/j.neunet.2006.10.001 17098563

[B27] Zhang J. Sclaroff S. (2013). Saliency detection: a boolean map approach, in 2013 IEEE International Conference on Computer Vision (Sydney, NSW: IEEE), 153–160. 10.1109/ICCV.2013.26

